# Clinical outcomes of sotrovimab in seronegative patients with severe COVID-19: A multicenter retrospective study

**DOI:** 10.1097/MD.0000000000046382

**Published:** 2026-01-02

**Authors:** Rubén Lobato-Cano, Laurine Prinet, Alberto Romero-Palacios, M. Paula Martín-Peral, Antonia M. de la Flor-Fuentes, Carmen Hidalgo-Tenorio, Paula Patricia García-Ocaña, Antonio Hidalgo-Castellón, Marina Murillo-Pineda, Manuel Corrales-Cuevas, Salvador López-Cárdenas

**Affiliations:** a Infectious Disease and Clinical Microbiology Unit, Hospital Universitario Jerez de la Frontera, Jerez de la Frontera (Cadiz), Spain; b Infectious Disease Unit, Hospital Universitario Virgen de las Nieves, Granada, Spain; c Infectious Disease Unit, Hospital Universitario Puerto Real, Puerto Real (Cadiz), Spain; d Instituto de Investigación e Innovación de Cádiz (INIBICA), Cadiz, Spain; e Department of Internal Medicine, Hospital Universitario Puerta del Mar, Cadiz, Spain; f Instituto de Investigación Biosanitaria de Granada (IBS-Granada), Granada, Spain; g Universidad de Cádiz, Cadiz, Spain.

**Keywords:** COVID-19, immunosuppression, inpatients, neutralizing antibodies, sotrovimab

## Abstract

The effectiveness of sotrovimab in severely immunocompromised inpatients with coronavirus disease (COVID-19) remains uncertain, particularly with the emergence of Omicron subvariants. This study aimed to evaluate the clinical outcomes of patients with severe COVID-19 who were seronegative for anti-S antibodies and who received sotrovimab. We conducted a retrospective cohort study at 4 teaching hospitals in the Andalusian Health System. Eligible participants were adults hospitalized with severe COVID-19, with negative anti-S serology, and treated with sotrovimab between December 2021 and March 2023. The primary outcomes were respiratory progression requiring advanced respiratory support and 28-day mortality rate. Secondary outcomes included the length of hospital stay and causes of readmission. Seventy-nine patients (58.2%) were male, with a median age of 72 years. Immunosuppression was mainly caused by hematologic malignancies (51.9%), solid organ transplantation (17.7%), and systemic autoimmune diseases (13.9%). The median time from symptom onset to sotrovimab infusion was 12 days and the median hospital stay was 13 days. The overall mortality rate was 36.7%, with 32.9% being directly attributable to COVID-19. No adverse reactions to sotrovimab were reported. In univariate analysis, older age and higher severity at admission were associated with disease progression. Patients were treated during periods dominated by Omicron subvariants BA.1, BA.2, BA.3, BQ.1, and XBB.1.5. The high mortality in this cohort underscores the extreme vulnerability of severely immunocompromised patients with COVID-19. These findings support further research into early and prolonged antiviral strategies, potentially combining agents, given the limitations of sotrovimab in the advanced disease stages.

## 1. Introduction

Neutralizing monoclonal antibodies (nAbs) against severe acute respiratory syndrome coronavirus 2 (SARS-CoV-2) target the spike protein at epitopes within and outside the receptor-binding domain and can be used for the prophylaxis and treatment of COVID-19.^[1]^ In ambulatory patients with risk factors and mild-to-moderate disease, nAbs reduced progression to hospitalization or death in randomized trials.^[2–4]^ Their role in hospitalized patients with severe COVID-19 is unclear, and some agents have been withdrawn from trials due to lack of efficacy.^[5,6]^ Nevertheless, benefits have been observed in patients with severe diseases that lack endogenous antibodies to SARS-CoV-2.^[7]^

Most nAbs have been developed against ancestral variants, and their neutralizing capacity has been variably reduced against newer subvariants, such as Omicron.^[8]^ Evidence of off-label use in hospitalized seronegative patients remains limited. Real-world data on the outcomes and safety in this population are essential to guide practice and inform therapeutic strategies.^[9]^ The objective of this study was to evaluate the clinical progression and safety in high-risk patients with severe COVID-19 who were seronegative for anti-S antibodies and treated with sotrovimab.

## 2. Materials and methods

### 2.1. Study design and setting

We conducted a retrospective, uncontrolled cohort study of patients hospitalized for COVID-19 between December 2021 and March 2023 at 4 public hospitals within the Andalusian Health System in Spain: University Hospital Jerez de la Frontera, University Hospital Puerto Real, University Hospital Puerta del Mar (Cadiz), and University Hospital Virgen de las Nieves (Granada). We adhered to the STROBE recommendations for reporting observational research.

### 2.2. Patients

The inclusion criteria were adults (≥18 years) with severe COVID-19 confirmed by reverse transcription polymerase chain reaction (RT-PCR) from nasopharyngeal samples, negative anti-S serology within the first week of admission, and treatment with a single 500 mg intravenous infusion of sotrovimab. Eligible patients had immunocompromising conditions, including hematologic malignancies, solid organ transplantation, or autoimmune diseases requiring immunosuppression. The exclusion criteria were incomplete drug infusion and the use of sotrovimab alone for the prevention of severe disease.

### 2.3. Data collection

Patients were identified through hospital pharmacy registries and study variables were retrieved from electronic medical records (Diraya, Andalusia, Spain). The collected data included demographics, comorbidities, baseline immunosuppressive therapy, vaccination status, COVID-19-specific treatments, length of hospital stay, and clinical outcomes. All relevant data are within the paper.

### 2.4. Outcomes and definitions

The primary outcome was respiratory progression, which was defined as the need for noninvasive ventilation, high-flow oxygen, invasive ventilation, extracorporeal membrane oxygenation, hemodynamic support, renal replacement therapy, or death within 28 days. Severity was classified using a modified World Health Organization ordinal scale for clinical progression (score ≥ 5).^[10]^ The secondary outcomes were 28-day mortality (COVID-19—attributable and all-cause), length of hospital stay, and readmission. Severe COVID-19 was defined by the World Health Organization criteria (clinical pneumonia plus respiratory rate > 30/min, severe distress, or SpO_2_ < 90% on room air).

### 2.5. Variant periods

Dominant Omicron subvariants during the study were determined from national surveillance reports^[11–13]^: BA.1 (week 51/2021–week 8/2022), BA.2 (weeks 9–23/2022), BA.5 (weeks 24–44/2022), BQ.1 (weeks 45/2022–week 4/2023), and XBB.1 (weeks 5–23/2023).

### 2.6. Statistical analysis

Categorical variables were described as frequencies and percentages and continuous variables as medians with interquartile ranges. Comparisons were performed using the chi-square test or Fisher exact test for categorical variables and Mann–Whitney *U* test for continuous variables. Two-sided *P*-values < .05 were considered statistically significant. Analyses were performed using SPSS, version 25.0 (IBM Corp, Armonk, NY).

### 2.7. Ethical considerations

This study was approved by the Research Ethics Committee of the Province of Cadiz, Spain. The requirement for informed consent was waived because of the retrospective design and the use of anonymized data.

## 3. Results

### 3.1. Patient characteristics

Seventy-nine patients were included: 46 (58.2%) were male, and the median age was 72 years (interquartile range [IQR], 65–79 years). The median age-adjusted Charlson comorbidity index score was 5 (IQR, 4–7). The major immunosuppressive factors were hematologic malignancies in 41 patients (51.9%), solid organ transplantation in 14 (17.7%; 10 renal, 1 renopancreatic, 2 lungs, and 1 hepatic), and systemic autoimmune disease in 11 (13.9%). The median time from symptom onset to sotrovimab infusion was 12 days (IQR: 8–22 days). The median hospital stay was 13 days (IQR: 13–26 days).

### 3.2. Outcomes

Respiratory progression (ordinal scale score ≥ 5) occurred in 24 patients (30.4%). Overall, 29 patients (36.7%) died, with 26 deaths (32.9%) directly attributable to COVID-19. No infusion-related adverse reactions were noted (Table 1). The secondary outcomes, including hospital stay and readmission, are presented in Table 2.

**Table 1:** Characteristics of severe COVID-19 inpatients with negative anti-S serology for SARS-CoV-2 treated with sotrovimab.

Variables	n = 79
Sex, n (%)	
Female/male	33 (41.8)/46 (58.2)
Age, median (P_25_–P_75_)	72 (65–79)
Comorbidity	
Charlson comorbidity index age-adjusted, median (P_25_–P_75_)	5 (4–7)
Diabetes, n (%)	20 (25.3)
Hypertension, n (%)	39 (49.4)
Obesity, n (%)	14 (17.7)
Hyperlipidemia, n (%)	35 (44.3)
Chronic kidney disease, n (%)	22 (27.8)
Solid organ neoplasia, n (%)	6 (7.6)
Hematologic neoplasia, n (%)	41 (51.9)
Chronic obstructive pulmonary disease, n (%)	8 (10.1)
Asthma, n (%)	4 (5.1)
Cirrhosis, n (%)	2 (2.5)
Autoimmune disease, n (%)	11 (13.9)
Organ solid transplant, n (%)	14 (17.7)
Primary immunosuppression, n (%)	3 (3.8)
Immunosupressive drug, n (%)	
Low-dose corticosteroids (<0.5 mg/kg)	24 (30.4)
Calcineurin inhibitors	12 (15.2)
mTOR inhibitors	2 (2.5)
Myeloablative chemotherapy	16 (20.3)
anti-CD20	28 (35.4)
Other	21 (26.6)
SARS-CoV-2 vaccination, n (%)	
Unvaccinated	9 (11.4)
Standard dose	23 (29.1)
Booster dose	47 (59.5)
Last dose > 5 months	40 (50.6)
Severity at admission, median (P_25_–P_75_)	
SpO_2_/FiO_2_	346 (124–429)
PaO_2_/FiO_2_ equivalence	319 (238–401)
Lab values, median (P_25_–P_75_)	
C-reactive protein (mg/L; ref 0–5)	119 (67–162)
Procalcitonin (ng/mL; ref <0.1)	0.15 (0.07–0.61)
Ferritin (ng/mL; ref 4.6–204)	748 (413–1537)
LDH (UI/dL; ref 125–220)	335 (236–451)
d-Dimer (ng/mL; ref 0–500)	657 (234–1304)
Lymphocytes (cell/µL; 1100–5000)	520 (320–1230)
SARS-CoV-2 treatment, n (%)	
Dexamethasone, 6 mg	51 (64.6)
Methylprednisolone, 2 mg/kg	21 (26.6)
Baricitinib	23 (29.1)
Tocilizumab	15 (19)
Convalescent plasma	2 (2.5)
Remdesivir	40 (50.6)
Nirmatrelvir/ritonavir	2 (2.5)
Clinical classification*, median (P_25_–P_75_)	
Day 0	2 (1–3)
Day 7	2 (1–5)
Day 14	1 (1–5)
Day 28	1 (1–7)
Mortality, n (%)	
Covid-19	26 (32.9)
Other causes	3 (3.8)
Covid-19 readmission, n (%)	3 (3.8)
Other causes readmission, n (%)	5 (6.3)
Symptoms before admission days, median (P_25_–P_75_)	9 (5–15)
Hospital stays days, median (P_25_–P_75_)	13 (13–26)
Days from symptoms to sotrovimab infusion, median (P_25_–P_75_)	12 (8–22)

* 1. No need for oxygen therapy or no increase in requirements in patients with home oxygen therapy (HOT); 2. Need for supplementary oxygen of 4 L/min or less via nasal cannula, or increased HOT requirements but less than or equal to 4 L/min; 3. Need for supplementary oxygen greater than 4 L/min with nasal cannula or use of Venturi masks; 4. Need for supplementary oxygen with non-rebreather mask; 5. Need for noninvasive ventilation or high-flow oxygen (i.e., high-flow nasal cannula); 6. Admission to intensive care unit due to need for invasive ventilation, extracorporeal membrane oxygenation, hemodynamic support, or renal replacement therapy; and 7. Death.

**Table 2:** Univariate analysis of severe COVID-19 inpatients with negative anti-S serology for SARS-CoV-2 who experience respiratory progression and/or death.

Variable	No progression, n = 55	Progression, n = 24	*P*
Sex, n (%)			
Female	22 (40)	11 (45.8)	.62
Male	33 (60)	13 (54.2)	
Age, median (P_25_–P_75_)	71 (63.5–75)	77 (65.2–80)	**.04**
Comorbidity			
Charlson comorbidity index adjusted for age, median (P_25_–P_75_)	5 (4–7)	6 (5–6.75)	.37
Diabetes, n (%)	11 (20)	9 (37.5)	.1
Hypertension, n (%)	26 (47.3)	13 (54.2)	.57
Obesity, n (%)	8 (14.5)	6 (25)	.26
Hyperlipidemia, n (%)	24 (68.6)	11 (31.4)	.85
Chronic kidney disease, n (%)	13 (23.6)	9 (37.5)	.2
Solid organ neoplasia, n (%)	4 (7.3)	2 (8.3)	.87
Hematologic neoplasia, n (%)	30 (54.5)	11 (45.8)	.47
Chronic obstructive pulmonary disease, n (%)	6 (10.9)	2 (8.3)	.7
Asthma, n (%)	3 (5.5)	1 (4.2)	.8
Cirrhosis, n (%)	7 (12.7)	4 (16.7)	.64
Autoimmune disease, n (%)	11 (20)	3 (12.5)	.34
Solid organ transplant, n (%)	10 (71.4)	4 (29.6)	.57
Primary immunodeficiency, n (%)	1 (1.8)	2 (8.3)	.42
Immunosupressive drug, n (%)			
Low-dose corticosteroids (<0.5 mg/kg)	19 (34.5)	5 (20.8)	.22
Calcineurin inhibitors	10 (18.2)	2 (8.3)	.26
mTOR inhibitors	1 (1.8)	1 (4.2)	.54
Myeloablative chemotherapy	14 (25.5)	2 (8.3)	.08
anti-CD20	21 (38.2)	7 (29.2)	.44
Other	16 (29.1)	5 (20.8)	.45
SARS-CoV-2 vaccination, n (%)			
Unvaccinated	7 (12.7)	2 (8.3)	.71
Standard dose	16 (29.1)	7 (30.4)	.9
Booster dose	28 (50.9)	15 (62.5)	.48
Last dose > 5 months	28 (56)	12 (54.5)	.9
Severity at admission, median (P_25_–P_75_)			
SpO_2_/FiO_2_	400 (170–440)	239 (118–387)	**.02**
PaO_2_/FiO_2_ equivalence	336 (291–410)	254 (146–364)	**.00**
Lab values, median (P_25_–P_75_)			
C-reactive protein (mg/L; ref 0–5)	114 (65–80)	125 (58–252)	**.04**
Procalcitonin (ng/mL; ref <0.1)	0.11 (0.06–0.35)	0.4 (0.08–1.54)	**.03**
Ferritin (ng/mL; ref 4.6–204)	854 (428–1502)	681 (297–2001)	.69
LDH (UI/dL; ref 125–220)	326 (237–422)	372 (236–487)	.34
d-Dimer (ng/mL; ref 0–500)	451 (101–1127)	1057 (556–3388)	**.04**
Lymphocytes (cell/µL; 1100–5000)	670 (365–1182)	425 (170–1200)	.67
SARS-CoV-2 treatment, n (%)			
Dexamethasone, 6 mg	39 (70.9)	12 (50)	.74
Methylprednisolone, 2 mg/kg	9 (16.4)	12 (50)	**.02**
Baricitinib	17 (30.9)	6 (25)	.59
Tocilizumab	7 (12.7)	8 (33.3)	.05
Convalescent plasma	1 (1.8)	1 (4.2)	.51
Remdesivir	30 (54.5)	10 (41.7)	.29
Nirmatrelvir/ritonavir	2 (3.6)	0 (0)	1
Symptoms before admission days, median (P_25_–P_75_)	9 (5–16)	9.5 (5–13)	.8
Hospital stays days, median (P_25_–P_75_)	13 (8–30)	13 (8–21)	.76
Days from symptoms to sotrovimab infusion, median (P_25_–P_75_)	11 (7–25)	13.5 (8–22)	.7

Bold values indicate statistical significance.

### 3.3. Analysis

Older age (median 77 vs 71 years, *P* = .04) and lower SpO_2_/FiO_2_ ratio at admission (median 239.5 vs 400, *P* = .02) were significantly associated with progression. Other variables were not statistically significant.

### 3.4. Variant periods

**Figure 1.**
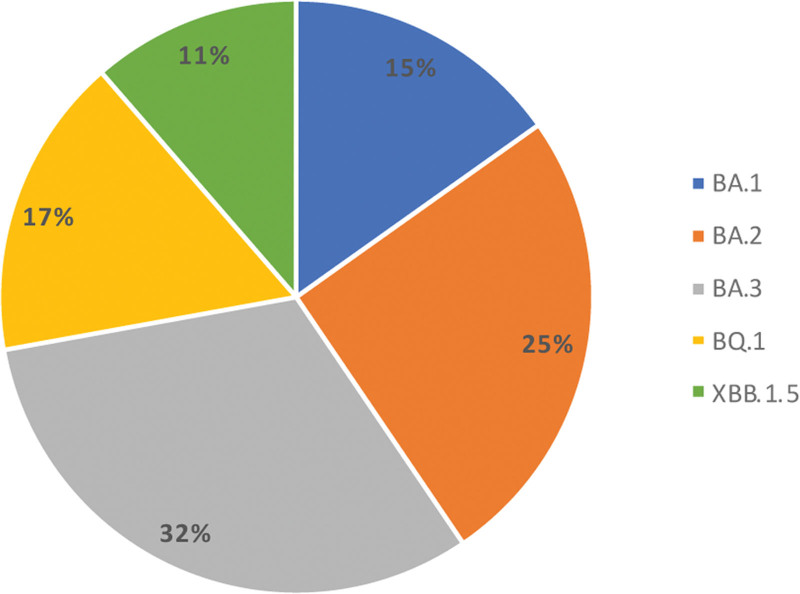
Temporal prevalence of SARS-CoV-2 subvariants in Spain among patients included according to reports from the RELECOV Technical Committee (Annual Report of the SARS-CoV-2 Sequencing Laboratory Network) and the Coordinating Center for Health Alert.

Patients were distributed according to dominant Omicron subvariants: BA.1 in 12 patients (15.2%), BA.2 in 20 (25.3%), BA.5 in 25 (31.6%), BQ.1 in 13 (16.5%), and XBB.1.5 in 9 (11.4%) (Fig. 1).

## 4. Discussion

Since the beginning of the pandemic, nAbs have been widely investigated as part of the therapeutic arsenal against COVID-19. Early administration has been shown to reduce the progression to severe disease, hospitalization, and mortality in patients with risk factors such as advanced age, diabetes, obesity, and cardiovascular disease.^[14]^ In hospitalized patients with severe COVID-19, the randomized evaluation of COVID-19 therapy (RECOVERY) trial demonstrated reduced mortality with casirivimab–imdevimab in seronegative patients,^[15]^ whereas the ACTIV-3/TICO trial found no efficacy of sotrovimab in a similar setting. Patients considered to be at a very high risk, particularly those with significant immunosuppression, have not been represented in pivotal clinical trials.^[4,16]^ Individuals with hematologic malignancies experience worse outcomes than other populations, with reported in-hospital mortality rates ranging from 21.3% to 36.5%.^[17,18]^ Observational studies suggest that early administration of nAbs in these subgroups may improve prognosis.^[19–21]^ In the European cohort of 1548 patients with hematologic malignancies, crude 30-day mortality decreased from 31.2% in the pre-vaccine era to 10.3% in the post-vaccine era, during which Omicron accounted for 68.7% of cases. In that study, nAbs were protective against 30-day mortality, both as monotherapy (hazard ratio [HR], 0.15; 95% confidence interval [CI], 0.07–0.31) and in combination with antivirals (HR, 0.40; 95% CI, 0.20–0.80), irrespective of disease severity at diagnosis.^[22]^ Among solid organ transplant recipients with mild-to-moderate COVID-19, a systematic review and meta-analysis found that sotrovimab was associated with reduced all-cause hospitalization (odds ratio, 0.29; 95% CI, 0.16–0.52) and 30-day mortality (odds ratio, 0.29; 95% CI, 0.03–0.64).^[23]^

The rapid emergence and spread of Omicron subvariants during 2022 led to the reduced in vitro neutralizing activity of sotrovimab against BA.2, BA.5, BQ.1, and XBB.1.5. This, together with the increased transmissibility, immune evasion, and virulence of the virus, complicates the comparison and interpretation of clinical studies across pandemic waves.^[9]^ Continuous monitoring of nAb activity is therefore required,^[24]^ and in March 2022, the US Food and Drug Administration restricted sotrovimab use based on reduced activity.^[25]^

Despite these in vitro findings, clinical efficacy appears to persist. A rapid review and meta-analysis of patients with mild-to-moderate COVID-19 treated with sotrovimab during Omicron-dominant periods showed reduced mortality and hospitalization, although no effect was demonstrated for disease progression or emergency department visits.^[26]^ In contrast, a retrospective cohort of 185 hospitalized patients with COVID-19 during BA.1, BA.2, or BA.4/5 circulation, adjusted with propensity score matching, found no difference in in-hospital mortality between those receiving sotrovimab (147 as monotherapy and 38 with remdesivir) and controls. These findings suggest no clear benefit of sotrovimab in this setting, although interpretation is limited by the lack of serologic data, incomplete vaccination status, and absence of precise information on the timing of infusion.^[27]^ One multicenter uncontrolled cohort of 32 patients, including 46.9% solid organ transplant recipients and 37.5% patients with hematologic malignancies, underwent active treatment. In that cohort, sotrovimab was associated with reduced clinical progression (12% vs 57.1%; *P* = .02) when administered within 14 days of symptom onset and in patients with PaO_2_/FiO_2_ ratios > 210, suggesting a potential benefit when given early to less severe cases.^[28]^ Although limited by the small sample size, this study was conducted during the circulation of delta and Omicron BA.1, against which sotrovimab retained its in vitro activity.

Another single-center retrospective study of immunosuppressed patients with poor vaccine responses reported that casirivimab–imdevimab, sotrovimab, or remdesivir used as monotherapy had protective effects (adjusted HR, 0.23; 95% CI, 0.08–0.65), whereas combination therapy was associated with the lowest risk of failure (adjusted HR, 0.06; 95% CI, 0.02–0.77). Limitations included administration restricted to the first 10 days after symptom onset and the exclusion of patients with severe hypoxemia (PaO_2_//FiO_2_ < 200, requiring high-flow oxygen or noninvasive ventilation).^[29]^

The most recent prospective multicenter study enrolled 245 immunocompromised patients treated with casirivimab–imdevimab, sotrovimab, or tixagevimab–cilgavimab during periods dominated by delta and Omicron variants. Among the 159 hospitalized patients, the overall mortality rate was 23%. The median time to viral clearance was 14 days (IQR, 7–22), but it extended to 63 days (95% CI, 57–69; *P* < .001) in those with resistance-associated mutations. These findings highlight both the high mortality and prolonged viral persistence in this population, and support the need to explore combined antiviral approaches.^[30]^ In our cohort, the burden of comorbidities and immunosuppressive conditions resulted in a considerable complexity and vulnerability. Factors such as advanced age, severity of hypoxemia at admission, and elevated inflammatory markers (C-reactive protein, procalcitonin, and d-dimer) were associated with clinical progression, consistent with prior reports,^[31]^ and remained key predictors of outcome. We found no association between the timing of sotrovimab infusion and prognosis; however, the median interval from symptom onset to treatment in both the progression and non-progression groups exceeded 10 days, suggesting that delayed administration may have limited potential benefits. In the recently published RECOVERY—sotrovimab trial conducted in UK hospitals during the Omicron-dominant period, 24% of the participants were severely immunocompromised. Sotrovimab showed no overall mortality benefit in hospitalized patients with COVID-19 pneumonia, but among those with high baseline SARS-CoV-2 nucleocapsid antigen levels, the 28-day mortality was lower (23% vs 29%),^[32]^ suggesting that targeted monoclonal antibody therapy may benefit selected high-risk subgroups.

We observed an association between methylprednisolone bolus use and clinical progression, most likely reflecting confounding by indication, as clinicians may have reserved this therapy in more severe cases. High-dose corticosteroids are no longer recommended; the RECOVERY trial demonstrated that dexamethasone 20 mg/d increased mortality compared to standard dosing at 6 mg/d in hypoxemic patients with COVID-19 (19% vs 12%; relative risk, 1.59; 95% CI, 1.20–2.10; *P* = .0012).^[33]^ Therefore, the current standard of care for severe COVID-19 is low-dose dexamethasone.

The potential impact of concomitant antiviral therapy should also be considered as combination regimens may influence outcomes.

Finally, this study was limited by its retrospective design, the absence of a control group, modest sample size, heterogeneity of immunosuppression, and lack of patient-level sequencing. Variant assignment relied on epidemiological surveillance, which may not fully capture the resistance patterns.

## 5. Conclusion

Despite sotrovimab administration, patients with severe COVID-19, immunosuppression, and negative anti-S serology experience high rates of progression and mortality. These findings suggest a limited clinical utility in advanced disease and underscore the need for early intervention strategies. Future research should prioritize controlled prospective studies in immunocompromised populations, evaluation of combination antiviral regimens, and assessment of efficacy against emerging variants to guide their optimal use.

## Acknowledgments

This manuscript was prepared with the assistance of artificial intelligence tools (ChatGPT-5, OpenAI, San Francisco, CA) for clarity, and Paperpal Manuscript Processing Report (Cactus Communications Services Pte Ltd, Singapore) for corrections. The authors reviewed and take full responsibility for the final content.

## Author contributions

**Data curation:** Rubén Lobato-Cano, Laurine Prinet, Alberto Romero-Palacios, M. Paula Martín-Peral, Antonia M. de la Flor-Fuentes, Carmen Hidalgo-Tenorio, Paula Patricia García-Ocaña, Antonio Hidalgo-Castellón, Marina Murillo-Pineda, Manuel Corrales-Cuevas.

**Formal analysis:** Rubén Lobato-Cano.

**Investigation:** Rubén Lobato-Cano.

**Methodology:** Rubén Lobato-Cano, Salvador López-Cárdenas.

**Supervision:** Salvador López-Cárdenas.

**Writing—original draft:** Rubén Lobato-Cano.

**Writing—review & editing:** Salvador López-Cárdenas.
